# Human Cytomegalovirus Entry into Dendritic Cells Occurs via a Macropinocytosis-Like Pathway in a pH-Independent and Cholesterol-Dependent Manner

**DOI:** 10.1371/journal.pone.0034795

**Published:** 2012-04-09

**Authors:** Fabienne Haspot, Amélie Lavault, Christian Sinzger, Kerstin Laib Sampaio, York-Dieter Stierhof, Paul Pilet, Céline Bressolette-Bodin, Franck Halary

**Affiliations:** 1 Unité Mixte de Recherche_S 1064 (ex643), Institut National de la Santé et de la Recherche Médicale, Institute for Transplantation/Urology and Nephrology (ITUN), Nantes, France; 2 Unité de Formation et de Recherche Médecine/Pharmacie, Université de Nantes, Nantes, France; 3 Centre Hospitalier Universitaire, Nantes, France; 4 Institute for Medical Virology and Epidemiology of Viral Diseases, Eberhard-Karls University, Tuebingen, Germany; 5 Center for Molecular Biology of Plants, Eberhard-Karls University, Tuebingen, Germany; 6 Unité Mixte de Recherche 791, Institut National de la Santé et de la Recherche Médicale, Nantes, France; 7 Equipe d’accueil 4271, Université de Nantes, Nantes, France; French National Centre for Scientific Research, France

## Abstract

Human cytomegalovirus (HCMV) is a ubiquitous herpesvirus that is able to infect fibroblastic, epithelial, endothelial and hematopoietic cells. Over the past ten years, several groups have provided direct evidence that dendritic cells (DCs) fully support the HCMV lytic cycle. We previously demonstrated that the C-type lectin dendritic cell-specific intercellular adhesion molecule-3-grabbing non-integrin (DC-SIGN) has a prominent role in the docking of HCMV on monocyte-derived DCs (MDDCs). The DC-SIGN/HCMV interaction was demonstrated to be a crucial and early event that substantially enhanced infection in *trans*, i.e., from one CMV-bearing cell to another non-infected cell (or trans-infection), and rendered susceptible cells fully permissive to HCMV infection. Nevertheless, nothing is yet known about how HCMV enters MDDCs. In this study, we demonstrated that VHL/E HCMV virions (an endothelio/dendrotropic strain) are first internalized into MDDCs by a macropinocytosis-like process in an actin- and cholesterol-dependent, but pH-independent, manner. We observed the accumulation of virions in large uncoated vesicles with endosomal features, and the virions remained as intact particles that retained infectious potential for several hours. This trans-infection property was specific to MDDCs because monocyte-derived macrophages or monocytes from the same donor were unable to allow the accumulation of and the subsequent transmission of the virus. Together, these data allowed us to delineate the early mechanisms of the internalization and entry of an endothelio/dendrotropic HCMV strain into human MDDCs and to propose that DCs can serve as a "Trojan horse" to convey CMV from entry sites to other locations that may favor the occurrence of either latency or acute infection.

## Introduction

Human cytomegalovirus (HCMV) can infect virtually any target cell of human origin; however viral transmission, systemic spread and proliferation occur in different cell types: epithelial cells, endothelial and hematopoietic cells and fibroblasts and smooth muscle cells, respectively (see [1] for review). HCMV initiates infection through a non-specific, low-avidity interaction with heparan sulfate proteoglycans (HSPGs; [2]). Then, higher avidity receptors, such as β2-microglobulin [3], HLA-B27 [4], annexin II [5], CD13 [6], [7], EGFR [8] or integrins [9] have been shown to promote stable attachment but to varying degrees. Most of these receptors have indeed been shown to play only minor roles [10], [11], [12] or even no role at all in HCMV capture and entry except for β1 integrins and to a lesser extent β3 integrins [13]. Indeed, β1 integrins have been shown to interact with HCMV particles and to further trigger intracellular signaling through a disintegrin-like motif of the envelope glycoprotein B (HCMV gB; [13]). Interestingly, we previously reported that HCMV gB is also a ligand for the C-type lectin receptor dendritic cell (DC) specific ICAM-grabbing non-integrin (DC-SIGN; [14]). We demonstrated that the DC-SIGN contribution was crucial because the blockade of DC-SIGN by antibody-mediated neutralization was necessary and sufficient to dramatically reduce (an 80 to 95% reduction) the binding of HCMV to the cell surface and to impair the productive infection of MDDCs by HCMV [14]. It now appears that HCMV likely uses distinct cell surface receptors with docking or entry-mediating properties, depending on the target cell.

Some animal viruses enter their host cells directly through penetration, i.e. release of the viral capsid in the cytosol after fusion between the viral envelope and the plasma membrane while the majority of them depend on endocytic uptake followed by viral penetration at the endosomal level. Endocytosis is a process by which a cell engulfs molecules that cannot pass through the plasma membrane. Endocytosis can occur via different mecanisms and most of them involve the uptake of fluid, solutes and small particles. Virus entry by endocytosis has been shown to involve clathrin, caveolin-1, lipid rafts, as well as Il-2, GEEC, ARF6, the flotillin pathway, macropinocytosis or phagocytosis (see [15] for review). Macropinocytosis is defined as an actin-dependent endocytic process involving plasma membrane ruffles which allowed internalization of fluid and particles in large uncoated endocytic vesicles (see [15] and [16] for review). The steps of HCMV internalization have only been partially elucidated because they appear to vary according to the HCMV strain tropism and the type of target cell. Although HCMV entry into fibroblasts, epithelial and endothelial cells has been shown to use distinct pathways [16], [17], [18], [19], nothing is known about the entry of HCMV into dendritic cells (DCs). It has been noticed that fibroblast infection is independent on actine polymerization whereas infection of endothelial cells and retinal pigment epithelial cells (RPE) is actin-dependent [17]. HCMV enters fibroblasts by direct fusion to the plasma membrane in a pH-independent manner [20]. Wild-type TR strain enters epithelial and endothelial cells by endocytosis, in this process the fusion requires endosome acidification [18]. Furthermore, it has been shown that HCMV can enter RPE cells by two distinct pathways depending on which cell type the virus is produced. A virus produced in epithelial cells will preferentially enter target RPE cells via fusion to the plasma membrane whereas virion generated in fibroblasts will enter by endocytosis [19]. A recent paper also demonstrates the importance of an intact UL128-131A region in HCMV monocyte infection and used electron microscopy to show an accumulation of HCMV virions in large vacuoles, suggesting that HCMV may not enter monocytes by direct fusion at the plasma membrane [21].

Immature DCs, which are known as professional antigen presenting cells (APCs), are characterized by their high endocytic capacity, which allows antigen uptake and processing (for review, see [22]). Alternatively, this fundamental property may be used by enveloped viruses to efficiently penetrate cells, thus partly preventing their lysosomal degradation and detection and neutralization by the immune system and allowing the release of viral genetic material very close to the target cell nucleus; the required location to initiate viral replication (for review, see [16]). Based on the ability of HCMV to interact with DC-SIGN [23], [24], [25], we endeavored to demonstrate how a highly endotheliotropic HCMV strain, VHL/E, that is also known to be leukotropic and even dendrotropic [21], [26], [27] is internalized and enter monocyte-derived DCs (MDDCs). Here we present evidence that VHL/E virion internalization and entry into MDDCs occur via a macropinocytosis-like pathway in a cholesterol-dependent and pH-independent manner. This entry pathway was shown to participate to the transmission of the virus from DCs to target cell also known as *trans*-infection.

## Materials and Methods

### Ethics Satement

Human fresh blood samples were obtained from the Etablissement Français du Sang, the French blood donor bank (EFS, Nantes, France). Human cells used in this study were prepared from healthy human volunteers. As a consequence no ethics statement is required for this work.

### Cells and Ragents

Peripheral blood mononuclear cells (PBMC) were isolated from whole blood by density centrifugation over Ficoll-Paque (Eurobio, Les Ulis, France). Different cell populations were enriched from PBMC by counterflow centrifuge elutriation using a Beckman Avanti J20 centrifuge equipped with a JE-5.0 rotor and a 40 mL elutriation chamber (Beckman Instrument Inc. Fullerton, USA). After introduction of the PBMC suspension, remaining platelets and red cells were elutriated first. The flow rate was increased in order to collect lymphocytes, and finally monocytes. Elutriated human monocytes were used to generate immature dendritic cells according to the protocol described by Sallusto and colleagues [28]; CD14+ cells (∼95% pure) were seeded at 1 × 10^6^ cells per ml in RPMI 1640-10% fetal calf serum with 2 mM glutamine supplemented with 100 ng/ml rhGM-CSF (Gentaur, Paris, France) and 20 ng/ml rhIL-4 (Cellgenix, Freiburg, Germany) and were cultured for five to seven days. Fully differentiated, immature DCs displayed the following standard phenotype: CD1a^+^, CD14^neg^, HLA-DR^+^, CD209^+^, CD80^low^, CD86^low^ and CD83^neg^ (as assessed using fluorescein isothiocyanate-conjugated or FITC-conjugated antibodies from BD Biosciences, CA, USA). Monocytes derived-macrophages (M1 type macrophages) were obtained from monocytes cultured during 5 days with 10ng/ml GM-CSF as described previously [29], [30]. Human foreskin fibroblasts (HFFs) were obtained from the Department of Immunology and Dermatology (Pr B. Dreno, CHU Nantes, France). HFFs were propagated in DMEM-10% fetal calf serum with 2 mM L-glutamine media. Recombinant soluble HCMV glycoprotein B (rec HCMV gB) was a kind gift from Biomérieux (France). DAPI, AlexaFluor 488-conjugated transferrin, AlexaFluor 488- or AlexaFluor 568-conjugated goat anti-mouse IgG mAb were obtained from Molecular Probes (Eugene, OR, USA). DMSO, 5-(N,N-dimethyl)-amiloride hydrochloride (DMA), cytochalasin D, bafilomycin A1, chlorpromazine, filipin, nystatin, methyl-β-cyclodextrin, ammonium chloride, rottlerin and Gö6983 were purchased from Sigma Aldrich (St. Louis, MO, USA) and Calbiochem (San Diego, CA, USA). Conjugated antibodies against CD14, CD1a, HLA-DR, CD80, CD83 and CD86 as well as purified anti-CD71 (transferrin receptor) and anti-CD209 (DC-SIGN, clone DCN46) were from Pharmingen (BD Biosciences, CA, USA). FITC-conjugated monoclonal antibodies against CD107b (LAMP2) and EEA1 were obtained from Pharmingen and Transduction Laboratories, respectively (BD Biosciences, USA). The unconjugated anti-HCMV glycoprotein B antibodies (clones HCMV37 or 2F12 used for confocal imaging and western blot detection, respectively) were purchased from Abcam (Cambridge, UK).

### Gradient Prification of HCMV Vrions

The endotheliotropic human HCMV strain VHL/E was used in this study and other published works [31]. The virus was propagated, purified and titered as described elsewhere [32], [33]. Briefly, gradient purification of VHL/E virions was performed with infectious supernatants from infected HFF cultures with approximately 100% late-stage cytopathic effects that were made cell free by centrifugation for ten minutes at 2,800 × *g*. Supernatants were then ultracentrifuged for 70 minutes at 80,000 × *g*. The pellets containing the virions and other particles were resuspended in 1 ml of phosphate-buffered saline (PBS) and were transferred onto a preformed linear glycerol-tartrate gradient (15–35% sodium tartrate and 30–0% glycerol in 0.04% sodium phosphate), which was then ultracentrifuged for 45 minutes at 80,000 × *g*. The virion-containing band was harvested with a syringe, and the virions were washed and pelleted by an additional ultracentrifugation step for 70 minutes at 80,000 × *g*. The pellet was resuspended in MEM5 and stored at −80°C until it was used for the infection experiments. The quality of the viral stocks was assessed by negative contrast transmission electron microscopy (Supplementary Figure S1). Usually, intact, enveloped virions accounted for more than 60–70% of physical particles after purification. Quantification of the virus was assessed with a plaque-forming assay on HFFs.

### DNA Etraction and Qantitative Ral-time PCR

Viral DNA was extracted using the Nucleospin® RNA virus kit (Macherey Nagel, France) according to the manufacturer’s instructions. A 67 bp fragment of the US8 viral gene was amplified using a quantitative real time PCR [34]. The oligonucleotides and probe used in this assay are as followed: forward 5′-GGCACCAAATGCAGAGTGAG-3′ (CMV1RGf), reverse 5′- AAGCCGTATTCCGTTTGCG-3′ (CMV2RGr) and 5′ FAM- TGGTCCAAGTCCGTGGGCACC-3′ TAMRA (CMVSRG). In order to exclude false-negative results, an internal amplification control was included in each reaction (TaqMan® Exogenous Internal Positive Control Reagents, IPC). CMV DNA quantitation achieved with a standard curve generated from 10-fold serial dilutions of a plasmid containing the viral target sequence. The HCMV DNA loads are expressed as absolute DNA copy numbers.

### Confocal Mcroscopy

Day 5 to 7 immature MDDCs were either treated or not with the appropriate drugs for 30 minutes at 37°C. Then, cells were infected with HCMV (MOI=2) or recombinant HCMV gB (2 µg/ml) for various periods of time. Thereafter, the cells were washed three times in PBS and were allowed to settle for at least 30 minutes at 37°C on poly-L-lysine-coated coverslips (overnight pretreated with 100 µg/ml in PBS at four degrees Celsius) then fixed for ten minutes with 4% paraformaldehyde (PFA) in PBS. Alternatively, cells were washed with a glycine-based acidic buffer (0.2 M, pH=2,8) immediately after the incubation step with HCMV to remove cell-associated virions. After washing with PBS and permeabilization at room temperature for ten minutes with PBS containing 0.2% Triton X-100, the cells were labeled with the appropriate primary and secondary antibodies as listed above and were mounted in Fluoromounting Medium (Dako). Nuclei were counterstained with DAPI as needed. The images were acquired in immersion (oil; magnification x63) on an SP5 confocal microscope (Leïca Microsystems, Germany).

### Transmission Eectron Mcroscopy

TEM was performed at the Electronic Microscopy Facility of the Federative Institute of Research 26 (IFR26, Nantes, France). Briefly, day 6 MDDCs were treated with medium alone or with inhibitors at the desired concentrations and were incubated with VHL/E (MOI=10) for varying time periods (30 minutes, two, six and 24 hours). Then, cells were washed with or without an additional acidic buffer inactivation step and were resuspended in the fixative solution (glutaraldehyde 2.5% v/v in Sorensen’s Phosphate Buffer at 0.1 M) for two hours and a half at 4°C. Cells were washed and post-fixed in 1% w/v osmium tetroxide for one hour at 4°C then were dehydrated in ethanol and embedded in an Epon resin mixture. Ultra-thin sections were double-stained using uranyl acetate and lead citrate. Finally, thin sections (60 to 70 nm) were cut on a Reichert Ultracut E microtome and were double-stained using uranyl acetate and lead citrate. Observation of the contrasted sections was done at 80 kV under a JEM-1010 transmission electron microscope (JEOL).

### Cytomegalovirus Ifection Ihibition Asay

One to two hundred thousands immature MDDCs per condition were treated with various doses of inhibitors for 30 minutes at 37°C before being incubated with the endotheliotropic HCMV strain VHL/E (MOI=2) for two additional hours at 37°C. Thereafter, non-internalized viral particles were removed by three washing steps (one with a low-pH glycine buffer) and infected cells were subcultured for 24 hours at 37°C. For each condition, a cell aliquot was kept to assess the cell viability by flow cytometry (using propidium iodide or DAPI); the drug concentrations used did not alter cell viability (data not shown). Cells were then allowed to adhere to poly-L-lysine-coated coverslips prior to be fixed and permeabilized with acetone and labeled for 30 minutes (37°C) with specific mAbs directed against immediate-early and early HCMV antigens (mAbs anti-I.E.A and −E.A, Argene Biosoft, Varilhes, France). A goat anti-mouse IgG polyclonal antibody conjugated to horseradish peroxidase (Dako) was subsequently applied for 30 minutes at 37°C. After each step, the slides were washed twice in PBS for five minutes. The presence of antigen was visualized by staining with aminoethyl carbazole (AEC; Argene Biosoft) for 15 minutes. After a ten minutes wash in dH_2_O, the slides were counterstained with hematoxylin (Sigma) and were mounted with glycerol/gelatin (Sigma).

Specimens incubated with isotypic antibodies (Dako) were used as negative controls. The slides were analyzed using a computer-based optical image analyzer (Eclipse E600 Nikon, Nikon Instruments, Inc., NY, USA). The analyses were performed at 20-fold magnification on four distinct fields situated 100 µm apart. The infection rate was then calculated as the mean value of the number of infected cells counted on the four distinct fields per condition divided by the mean value of the total number of counted cells and multiplied by 100 (a semi-automated counting was done using the ImageJ software).

### Subcellular Factionation Assay

To determine the location of the internalized HCMV virions in MDDCs, we adapted a subcellular fractionation assay from Segura and colleagues [35]. Briefly, immature MDDCs (0.7–10×10^7^ cells per condition) were incubated with VHL/E (MOI=10) for two hours at 37°C. After one acidic buffer inactivation and three additional washes in PBS, the cells were homogenized with a cell-cracker (Kimble Chase Life Science, NJ, USA) in homogenization buffer (PBS, 0.25 M sucrose, 10 mM Tris, 1 mM EDTA supplemented with protease inhibitors purchased from Roche, pH 6.8). Postnuclear supernatant (PN) was prepared by centrifugation (1,000 × *g* for ten minutes) and was loaded on top of a 10% Percoll (GE Healthcare) solution in homogenization buffer. After ultracentrifugation (50,000 × *g* for 45 minutes), one mL fractions were collected. The top three fractions (early endosomes) were pooled and concentrated by ultracentrifugation (130,000 × *g* for one hour). The bottom two fractions were pooled and loaded on top of a 45% Percoll solution in homogenization buffer. After ultracentrifugation (50,000 × *g* for 45 minutes), the top three fractions (late endosomes) and the bottom two fractions (lysosomes) were pooled and concentrated by ultracentrifugation (130,000 × *g* for one hour). When needed, the collected fractions were pooled and concentrated onto Centricon-3 filtration devices (cut-off 10 kDa; Millipore, Bedford, MA, USA) and were frozen at −80°C until use or were instantly analyzed with western blot.

### Western Blot Analysis

Protein contents of subcellular fraction were quantified by BCA assay (Sigma) according to the manufacturer’s instructions. In the absence of a conserved reference protein in all subcellular fractions obtained after cell fractionation equal amounts of total protein were loaded on a 10% SDS/PAGE gel (Pierce Biotechnology, Rockford, IL, USA) under non-reducing conditions and transferred onto nitrocellulose membranes. The membrane were stained with serum against MCP (a kind gift from Dr. Wade Gibson, Pharmacology and Molecular Sciences, Johns Hopkins University, Baltimore, MD, USA) or mAbs against human EEA1, LAMP-2 and HCMV gB (clone 2F12) purchased from Abcam. A horseradish peroxidase (HRP)-conjugated anti-mouse IgG antibody (Pierce) was used for protein detection.

### Trans-infection Experiment

On Day 0, frozen monocytes from healthy human volunteers were thawed and differentiated for five days toward MDDCs and M1-typed macrophages (see the "Cells and reagents" part of the Material and Method section for more information). On day 5, another vial of monocytes from the same healthy volunteer was thawed and all three cell types were cultured for 30 minutes with or without Bafilomycin A1. Then, cells were infected with HCMV (MOI=2) for two hours and afterwards extensively washed with PBS or with a low-pH buffer. Infected cells were then co-cultured in close contact with fibroblast monolayer. After fourty-eight hours, fibroblasts were processed and labeled as described above (*Cytomegalovirus infection inhibition assay)* to evaluate the trans-infection by HCMV-loaded cells.

### Statistical Analysis

Statistical analysis were performed using the non-parametric Mann-Whitney test with Prism GraphPad software; p values less than 0.05 were considered to be significant.

## Results

### HCMV Internalization by MDDCs is Dependent on Cytoskeleton Remodeling

**Figure 1:**
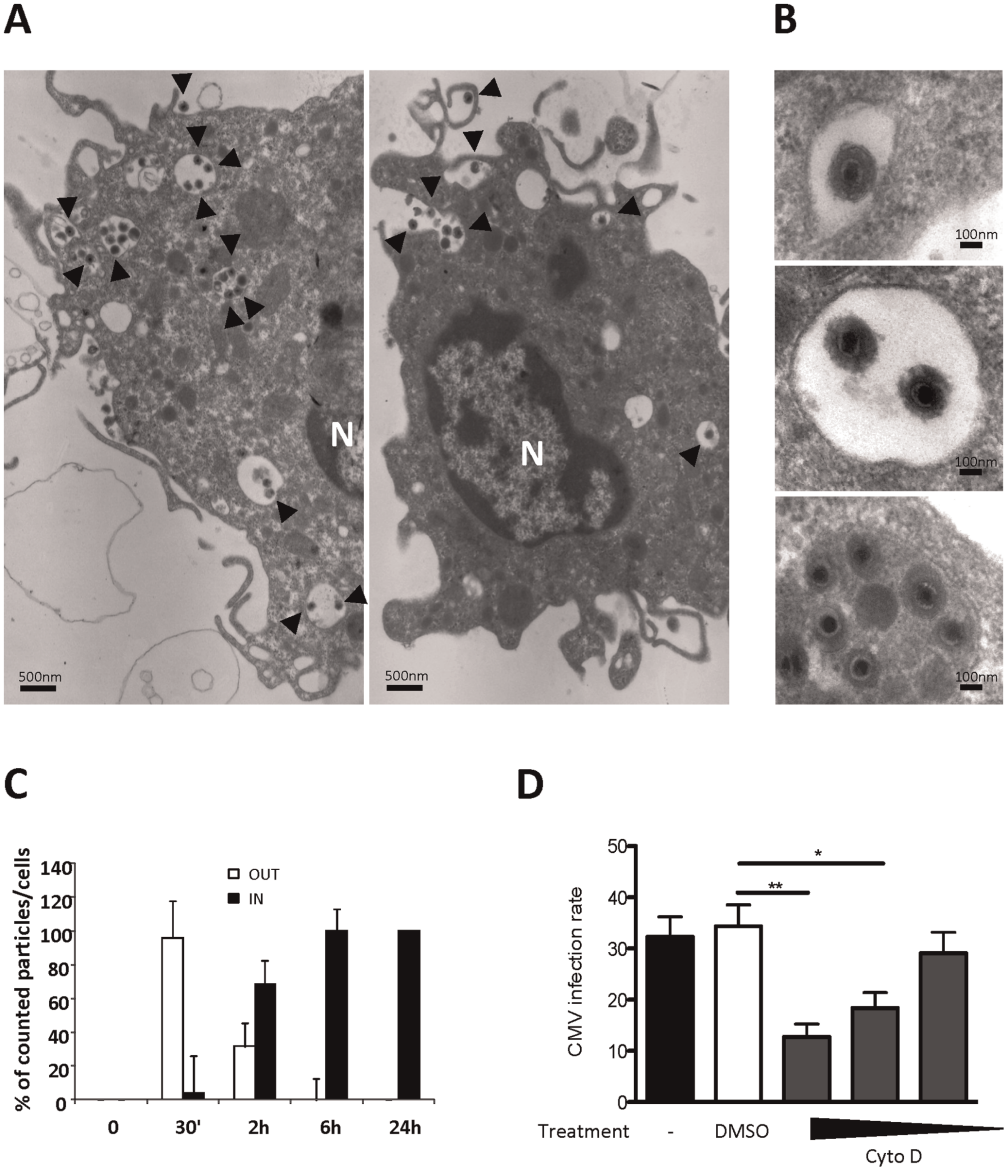
CMV internalization into large uncoated vesicles in MDDCs is an active process that requires actin cytoskeleton polymerization. A) Transmission electron microscopy (TEM) picture is shown of day 6 immature MDDCs incubated with HCMV (VHL/E; MOI=10) for two hours then extensively washed with PBS. A scale bar is indicated (magnification x7,500). N=nucleus. B) Close-ups of different pictures are represented. Black arrows indicate HCMV virions. A scale bar is indicated for each picture. Cells were prepared as described in 1A. C) Kinetic analysis of HCMV internalization in MDDCs. The data represent the quantification of the mean number of particles per cell from the TEM pictures (n≥20 independent cells) of viral particles (VHL/E; MOI=10) immobilized at the plasma membrane (OUT, white bars) or internalized into vacuoles (IN, black bars) as function of time (30 minutes, two, six and 24 hours incubations). Cells were extensively washed with PBS after incubation with HCMV. These results are representative of two distinct experiments. D) MDDCs were pre-incubated with drugs (cytochalasin, cytD: 50-5-0.5 µM) or vehicle (DMSO: 1/100) prior to be infected with HCMV (MOI=2) for two hours. Cells were extensively washed then subcultured for 48 hours. The cells were coated onto poly-L-lysine-coated slides, fixed and permeabilized with acetone and were stained with mAbs against anti-IE and –E antigens (Argene Biosoft, France). Four distinct fields were digitalized and analyzed with ImageJ software to determine the percentage of I.E.A./E.A.+ MDDCs. n=6 independent experiments with eight different donors in total.

To identify the point of the entry of HCMV particles into MDDCs, we analyzed MDDCs after short-term HCMV infection (up to two hours) by transmission electron microscopy (TEM). We first noted that HCMV particles were partly surrounded by cell membrane ruffles, suggesting that actin polymerization is needed to allow membrane remodeling and the engulfment of HCMV virions (Figure 1A). We also observed that the vesicle contents other than the HCMV virions were either transparent or moderately dense to electrons, suggesting that virions may accumulate in early endocytic vesicles of varying phenotypes and most likely with distinct properties (Figure 1B). A few dense bodies were also observed mixed with infectious particles but might behave during entry as intact particles due to their envelopes similar to those of infectious virions. The kinetics of MDDC infection allowed us to quantify that 70% of the HCMV virions were located in yet unknown vacuoles inside the cells, and 30% of the virions were still bound to the plasma membrane (Figure 1C). By six hours post-infection, all of the virions detected were localized inside the cells (Figure 1C). To test whether HCMV internalization is an actin-dependent process, we used cytoskeleton pharmacological inhibitors that are able to prevent actin polymerization and analyzed the effects of these inhibitors on the CMV infection of MDDCs as a downstream event of the viral entry. Day six MDDCs were pretreated with increasing concentrations of actin inhibitors (or vehicle controls) for 30 minutes prior to be infected with HCMV (MOI=2) for two hours. After extensive washes followed by 48 hours of culture, the cells were stained for HCMV IE/E nuclear antigens and counterstained with hematoxylin to allow total cell counting. Simultaneously, cell viability was assessed for each condition by flow cytometric analysis (DAPI or propidium iodide exclusion; data not shown). When pretreated with cytochalasin D (5 µM) (Figure 1 D) or latrunculin A1 (10 µM) (data not shown), infection of MDDCs by HCMV was significantly impaired as compared to DMSO-treated (vehicle) or to non-treated cells (Figure 1D), confirming that actin remodeling is necessary for HCMV engulfment into MDDCs.

### Enveloped HCMV Particles Accumulate in Large Vesicles with Endosomal Features in MDDCs before they Undergo Penetration by Uncoating

**Figure 2:**
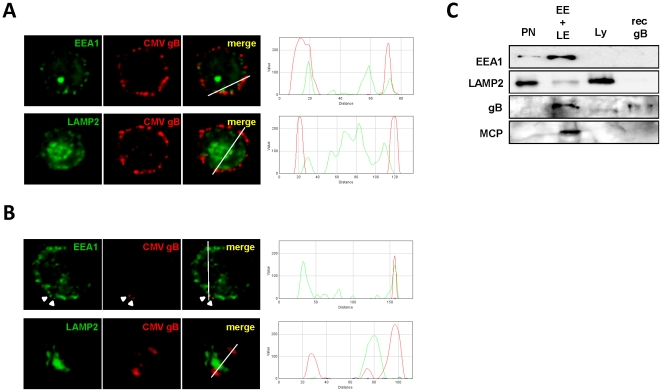
Internalized HCMV virions partially co localize with EEA1 and not with LAMP2. Confocal imaging was performed on immobilized immature MDDCs incubated for two hours on ice with the VHL/E strain (MOI=2) and subsequently cultured at 37°C for three hours (A) and 24 hours (B). Cells were then fixed/permeabilized and immunostained as indicated above each column with anti-EEA1 or -LAMP2 antibodies (green) and with an anti-HCMV gB (red). Images were obtained on a 510 LSM Meta (Zeiss, Germany). Single confocal planes are presented. In merged panels, green and red staining intensities were directly analyzed along a virtual section indicated by a thin white line with the ImageJ RGB profiler plugin. The results are displayed in graphs on the right (X-axis=distance in pixels; Y-axis=fluorescence intensity). White arrowheads indicate small CMV aggregates or isolated particles. C) Between 7 and 10 x 10^7^ HCMV-infected MDDCs (VHL/E; MOI=10) were subjected to three consecutive subcellular fractionation steps on sucrose and Percoll gradients to harvest early and late endosome-enriched and lysosome-enriched fractions. Here early endosomes (EE) and late endosomes (LE) enriched fractions were first pooled (EE+LE) and the EE+LE and the lysosomes (Ly) enriched fractions were concentrated before being used in a western blot analysis using anti-EEA1, LAMP-2, HCMV gB and MCP antibodies. Recombinant HCMV gB (Biomérieux, France) was used as a positive control. The molecular weight of the gB is 160 kDa and 153 kDa for the MCP. PN means post nuclear fraction.

We sought to identify the nature of the large vesicles containing the internalized HCMV particles we described previously. To that purpose we infected immobilized MDDCs with the VHL/E strain at 37°C for three and 24 hours. Then, cells were fixed/permeabilized and labeled to characterize the location of HCMV (red staining) into different types of organelles identified by the early endosome antigen 1 (EEA1) and the LAMP2 markers (green staining), which are preferentially expressed by early and late endosomes or lysosomes, respectively. The pictures obtained with confocal imaging are displayed in Figure 2A and 2B and were analyzed with the ImageJ RGB profiler pluging. For both time points, we observed some co-localization of HCMV with EEA1, suggesting that the virus was internalized into large endocytic vesicles matching the features of early and late endosomes. Surprisingly, we were unable to observe colocalization between HCMV and the LAMP2 staining, at any time points.

We performed subcellular fractionation to extract and enrich the early/late endosomes (EE+LE) and the lysosomes (Ly). Separation quality was assessed by western blot using antibodies against EEA1, LAMP2. We performed the experiment using HCMV-infected MDDCs (two hours, MOI=10). Firstly, we looked for the presence of two viral proteins, the envelope protein gB (160 kDa) and MCP major capsid component (153 kDa), within the tested fractions (Figure 2C). Both viral antigens were found at the expected molecular weight (153 kDa MCP and 160 kDa gB) only in the early and late endosome-containing fraction. Neither MCP nor gB could be evidenced in the PN fraction. This was most likely due to a lack of sensitivity of the western blot assay since to obtain the endosomes- or lysosomes-enriched fractions (ca. 200 µl) the PN fraction (7 ml) has to be 25- to 35-fold concentrated. In the Ly, the gB was detected as a protein of lower molecular weight as compared to the recombinant gB. In addition, the relative amount of gB in the Ly fraction appeared to be diminished as compared to the EE+LE fraction since equal amount of protein were loaded on the gel. This suggests that degradation of viral gB may have been initiated. Moreover, no MCP was detected in the lysosome-enriched fraction. Secondly we quantified the HCMV genome copy number in subcellular fractions by quantitative PCR (Supplementary Figure S2). HCMV genomic DNA could be detected in fraction containing early and late endosomes as well as lysosomes. These results go in line with our co-localization study described previously (Figure 2A and 2B) and may suggest that the viral penetration could occur before the lysosomal stage.

**Figure 3:**
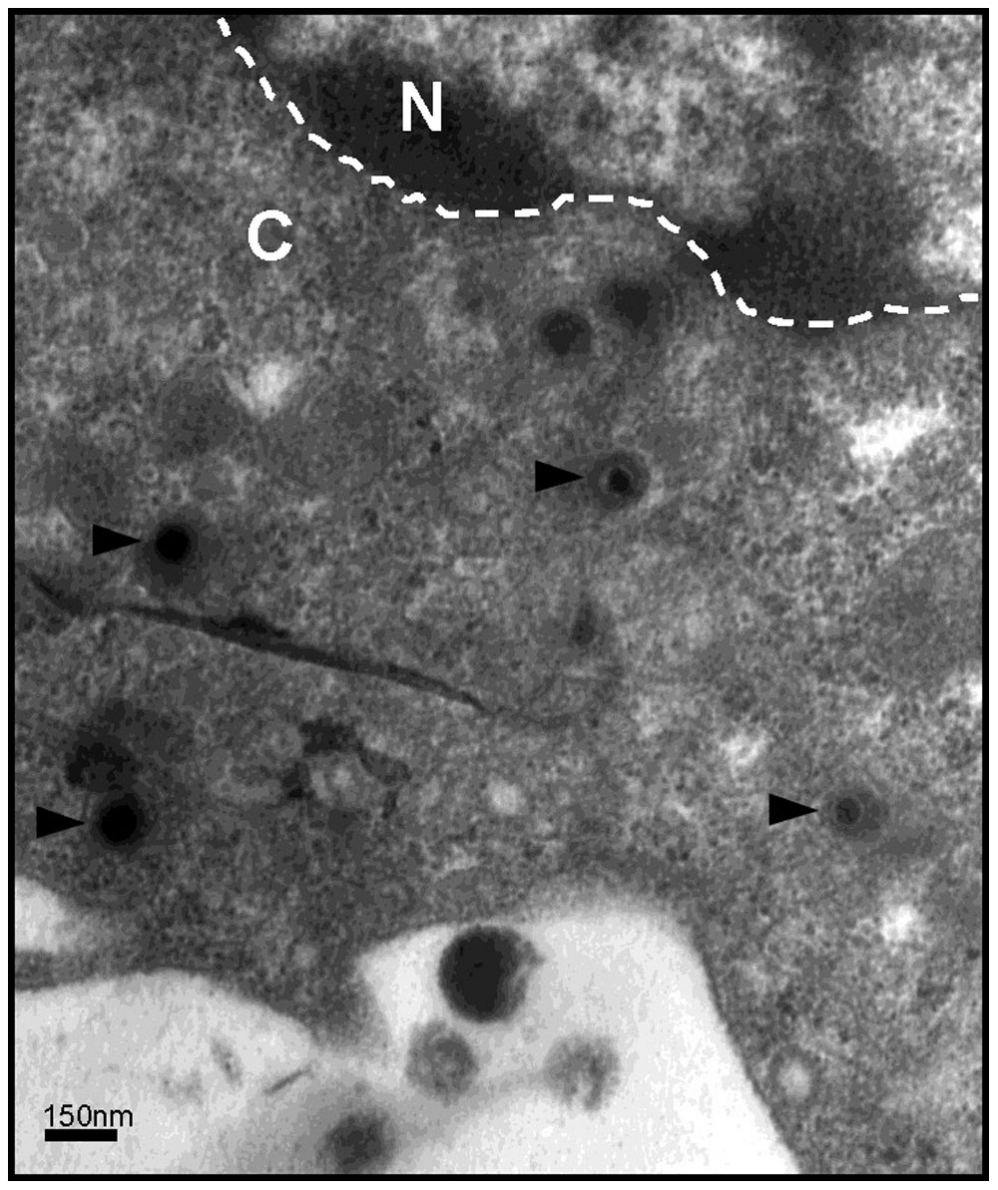
Uncoating of internalized particles results in naked capsids in cytoplasm of infected MDDCs. Transmission electron microscopy (TEM) picture of a Day 6 immature MDDC incubated with HCMV (VHL/E; MOI=10) for six hours then extensively washed with PBS. Naked capsids in the cytoplasm are marked by arrowheads. A scale bar is indicated in the lower left part of the micrograph (magnification x200,000). A white dashed line indicates the nuclear envelope. N= nucleus; C= cytoplasm.

In order to fully demonstrate that internalized HCMV particles do infect MDDCs we carefully analyzed ultrathin sections of MDDCs inoculated for six hours with VHL/E particles to evidence the presence of uncoated capsids in MDDCs cytoplasm. Thus, in a substantial number of cells we simultaneously observed a vast majority of intact virions within vesicles and structures resembling to uncoated capsids in the cytosol (Figure 3). Remarkably these naked capsids were usually found at the proximity of the nuclear envelope (Figure 3). These observations strongly suggested that at least some of the internalized HCMV particles underwent penetration by uncoating and release of the capsid in the cytoplasm before they may gain access to nuclear pores to inject their DNA into the nucleus.

Finaly, we compared the internalization of whole HCMV particles and virus-free recombinant soluble gB, both of which are able to bind DC-SIGN [14]. We observed that whereas recombinant gB was completely conveyed to lysosomes after only 15 minutes (complete colocalization with LAMP2), whole HCMV virions never colocalized with the lysosomal marker LAMP2 (Supplementary Figure S3A and B), suggesting that they could be sequestered into the previously described EEA1+ vesicles. Although the difference in terms of size and molecular orientation and/or multimerization between HCMV particles and virus-free recombinant soluble gB might explain this observation, we would like to highlight the fact that the pH of the LAMP2-positive endosomes did not dramatically impair the staining of HCMV gB (no epitope damage) at least for short time periods (up to 30 minutes, data not shown). When analyzing HCMV-infected MDDCs by TEM, we observed intact particles embedded in vesicles with low-density contents (Figure 1B). Taken together, these results provide strong evidence that HCMV particles are sequestered into early endosomes as intact virions.

### HCMV Internalization into MDDCs Occurs Via a Macropinocytosis-like Pathway

**Figure 4:**
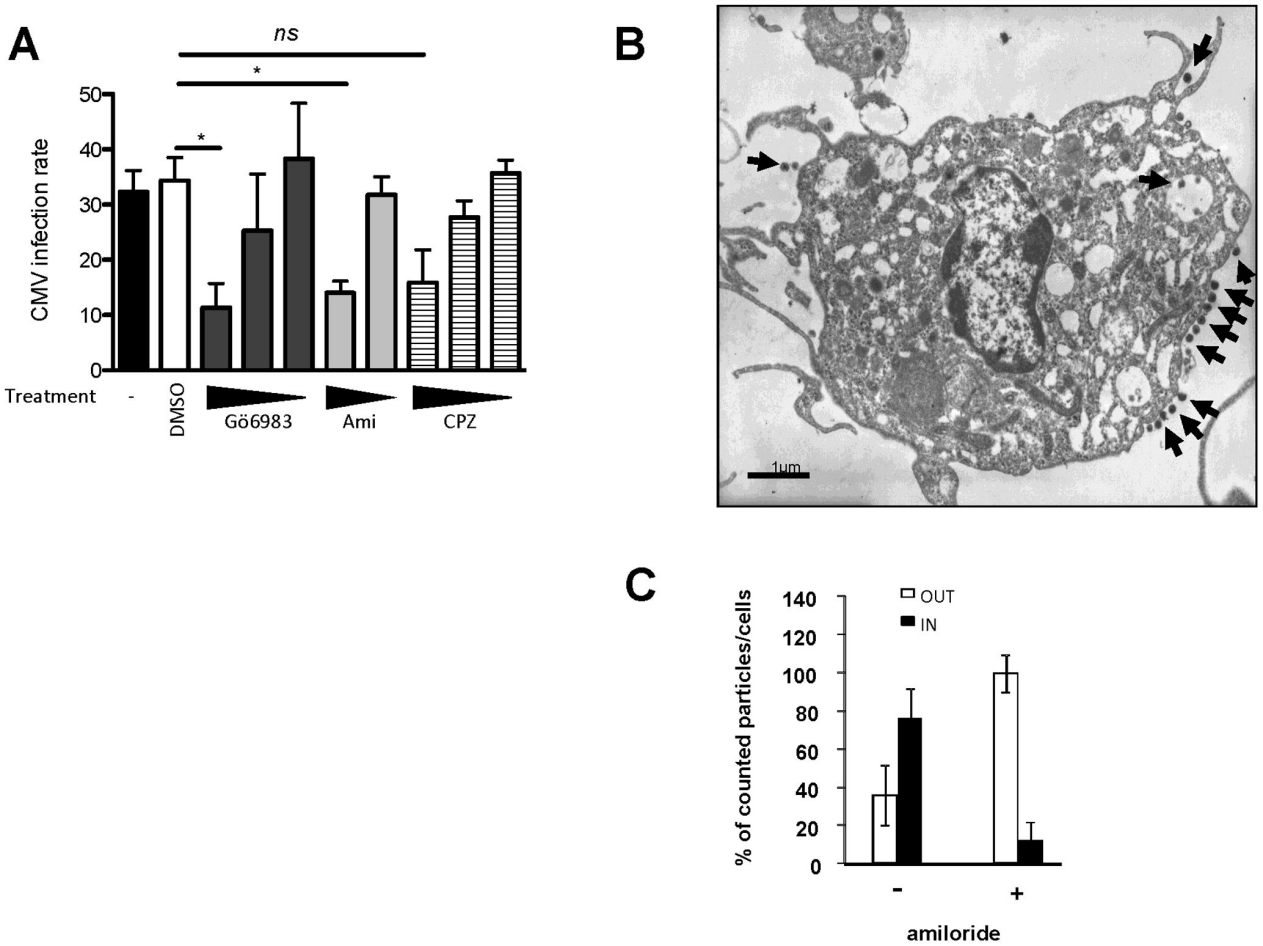
HCMV internalization into MDDCs is impaired by macropinocytosis inhibitors. A) MDDCs were pre-incubated with drugs blocking macropinocytosis (Gö6983∶ 13, 7.3, 3.75 nM and amiloride, Ami: 100, 20 µM) or clathrin-mediated endocytosis (chlorpromazine, CPZ: 30, 6, 1.2 µM) or with vehicle (DMSO, 1/100) prior to be infected with HCMV (MOI=2) for two hours. Cells were extensively washed then subcultured for 48 hours. The cells were then prepared and analyzed as described in the legend for Figure 1D. For Gö6983, n=2 independent experiments with 4 different donors in total, for Ami and CPZ n=5 independent experiments with 7 different donors in total. ns: not significant (p=0,0733) B) TEM picture of (500µM) amiloride-treated MDDCs incubated with HCMV (VHL/E; MOI=10). Black arrows indicate HCMV virions. C) Quantification of infectious HCMV particles by TEM immobilized at the plasma membrane (OUT, white bars) or internalized into vacuoles (IN, black bars) of (500 µM) amiloride-treated MDDCs incubated for two hours with VHL/E (MOI=10) (n=6 cells per conditions). The results are displayed as the median values of the percentage (±SD) of plasma membrane-associated and internalized HCMV particles.

HCMV is one of the largest animal viruses (150–300 nm in diameter). Considering the size of HCMV particles and based on our own observations, we concluded that most of the endocytic pathways (see [36] for review) except for macropinocytosis could not be involved in the HCMV internalization into MDDCs. Indeed, other herpes viruses such as Epstein-Barr and Herplex simplex virus were shown to be endocytosed in large uncoated vesicle and involving mechanisms suggesting a role for macropinosomes [37]. When we analyzed MDDCs two hours post-infection by high magnification TEM, we noticed HCMV particles in forming or closed large uncoated vesicles (Figure 1A-B). We used pharmacological inhibitors of macropinocytosis to treat MDDCs before infection with HCMV. Amiloride, a well-known Na^+^/H^+^ exchanger inhibitor, has also been widely used as a selective blocker of fluid-phase endocytosis (FPE) and macropinocytosis in endothelial cells and MDDCs [38]. In addition to amiloride, we tested the effect of the PKC inhibitor Gö6983 on the infection of MDDCs by HCMV. As shown in Figure 4A both amiloride (100 µM) and GÖ6983 (13 nM) significantly reduced the infection rate in a dose-dependent manner. We obtained the same results with the rottlerin (Suplementary Figure S4). We confirmed that the concentration of amiloride (100 µM) used was efficient at blocking the macropinocytosis of 70,000 Da FITC-dextran in MDDCs (data not shown). Furthermore, TEM analysis of amiloride-treated MDDCs infected with HCMV clearly showed that all particles but one were docked at the plasma membrane (Figure 4B). Of note, despite the documented efficacy of amiloride in our study, amiloride treatment promoted only minor changes in the number and size of filipodia-like structures in MDDCs as compared to previous published results obtained with epithelial cells [39], [40], [41]. The quantification of this observation showed that impairment in the macropinocytosis capability of MDDCs results in a strong defect in the entry of HCMV into the cells (Figure 4C). Finally, chlorpromazine, a clathrin-dependent endocytosis inhibitor, had no effect on the infection rate, even at the highest concentration (30 µM) (Figure 4A). Altogether, these results show that HCMV particles are internalized into MDDCs by a macropinocytic-like pathway.

### HCMV Internalization into MDDCs Occurs in a Cholesterol-dependent Manner

**Figure 5:**
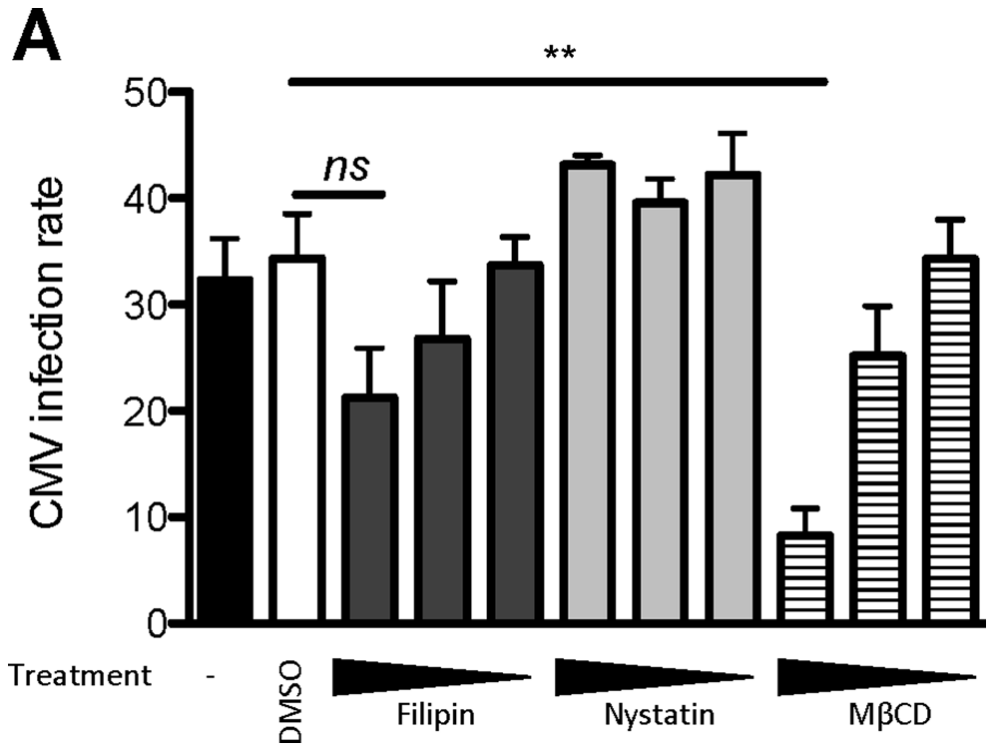
Cholesterol depletion is detrimental to the HCMV entry into MDDCs. A) Cells were pre-incubated with filipin (7.66, 1.5, 0.3 µM), nystatin (21.2, 4.3, 0.85 µM) or methyl-β-cyclodextrin (MβCD; 5, 1, 0.2 mM) or with vehicle (DMSO, 1/100) and were processed as described in the legend for Figure 1D. For nystatin, n= 2 independent experiments with 2 different donors in total; for Filipin and MβCD n=4 independent experiments with 6 different donors in total. *ns*: not significant (p=0,0535).

We previously demonstrated that DC-SIGN is necessary for HCMV docking on the MDDC plasma membrane [14]. Moreover, several studies have shown that DC-SIGN is organized in cholesterol-enriched microdomains on the plasma membrane of immature, living MDDCs [42], [43], [44]. Thus, we hypothesized that HCMV internalization could involve cholesterol-enriched lipid rafts. To confirm our hypothesis, we disrupted the cholesterol-containing microdomains of MDDCs with increasing doses of methyl-β-cyclodextrin (MβCD), a methylated cyclic oligosaccharide that is able to solubilize cholesterol and remove it from the plasma membrane [45]. The cells were then cultured with HCMV for two hours and the infection rate was calculated as described earlier. We observed that cholesterol depletion significantly decreased the infection of MDDCs as compared to cells treated with vehicle proving that cholesterol-enriched microdomains are essential for the internalization of HCMV into these cells (Figure 5A). To ensure that other types of microdomains were not involved in the endocytic process leading to HCMV infection, we also tested whether filipin and nystatin, both of which complex with cholesterol in lipid rafts but do not extract it, inhibited HCMV infection in MDDCs. Neither filipin nor nystatin had an effect on the infection rate compared to vehicle-treated cells (Figure 5A). Those results were rather unexpected since filipin and nystatin do disturb cholesterol-enriched microdomains. However, they are in accordance with previous studies reporting that filipin and nystatin may not efficiently inhibit cholesterol-dependent endocytosis and even in some cases may enhance it [46], [47]. These results demonstrated HCMV internalization is also dependent on these cholesterol-enriched microdomains.

### Infection of MDDCs with HCMV does not Depend on Endosomal Acidification

**Figure 6:**
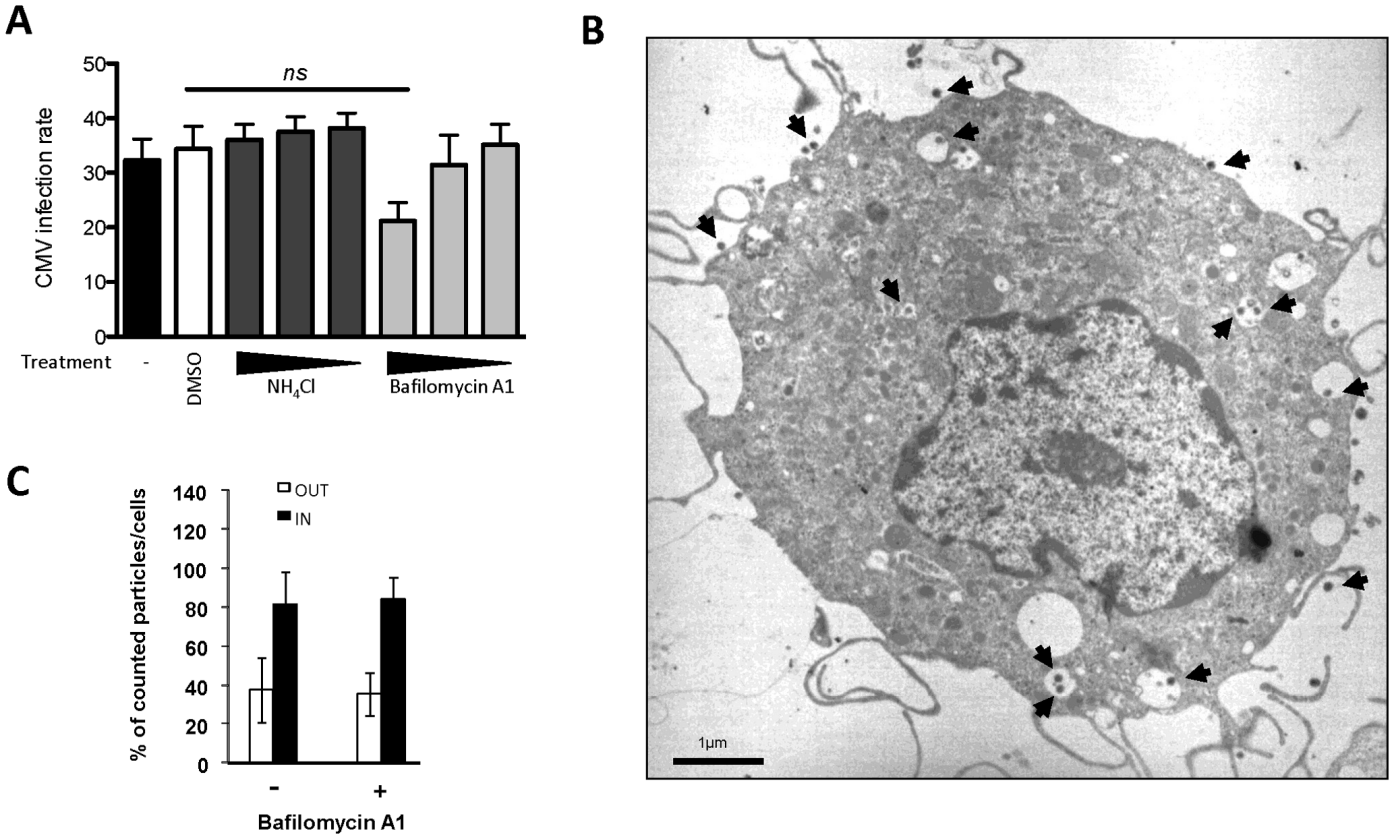
Endosomal pH neutralization does not inhibit HCMV internalization or MDDC infection. A) Cells were pre-incubated with NH_4_Cl-containing buffer (50, 5, 0.5 mM) or bafilomycin A1 (320, 32, 3.2 nM) and compared to the vehicle (DMSO; 1/100). The cells were processed as described in the legend for Figure 1D. For NH_4_Cl, n= 3 independent experiments with 3 different donors in total, for bafilomycin A1 n= 6 independent experiments with 8 different donors in total. ns: not significant (p=0,0939). B) TEM picture of (50 nM) bafilomycin A1 treated MDDCs incubated with HCMV (VHL/E; MOI=10). Black arrows indicate HCMV virions. C) Quantification of infectious HCMV particles by TEM immobilized at the plasma membrane (OUT, white bars) or internalized into vacuoles (IN, black bars) of (50 nM) BafA1-treated MDDCs incubated for two hours with VHL/E (MOI=10) (n=5-6 cells per condition). The results are displayed as the median values of the percentage (±SD) of plasma membrane-associated and internalized HCMV particles. These results are representative of two distinct experiments.

Among viruses that infect mammalian cells, some require endosomal acidification to efficiently release their capsid into the cytoplasm. This step is commonly defined as the viral penetration. However, in some cases, such as for Herpes simplex Virus 1 (HSV-1), Ebola or the SARS coronavirus, a low pH is not sufficient to induce fusion between the viral envelope and the endocytic vesicle membrane [36], [38]. Recently, it was reported that endotheliotropic HCMV strains, which are also known to productively infect MDDCs, use a pH-independent, but very fast, endocytic pathway of entry into endothelial cells [1]. Based on these observations, we wondered whether the infection of MDDCs by HCMV was dependent on a decrease in the endosomal pH. To address this question, we pretreated immature MDDCs with an ammonium chloride buffer (NH_4_Cl) or bafilomycin A1 (BafA1), which stabilize the endocytic pH by buffering the pH or blocking the V-ATPase responsible for the endosomal pH decrease, respectively, before incubation with HCMV. Ammonium chloride or bafilomycin A1 pretreatments had no effects on the rate of MDDC infection in comparison to the vehicle alone (Figure 6A). Based on TEM, we observed that pretreatment of MDDCs with BafA1 (50 nM) did not impair HCMV internalization (Figure 6B) or the subsequent steps of the infection process as described previously in this paper. Indeed, quantification of the HCMV particles outside and inside the bafilomycin-A1-treated MDDCs and the non-treated MDDCs revealed no difference (Figure 6C). These results strongly suggest that endosomal acidification is not necessary for the entry of HCMV into MDDCs and the infection of these cells.

### Intracellularly-stocked HCMV Virions can Mediate Trans-Infection by MDDCs

**Figure 7:**
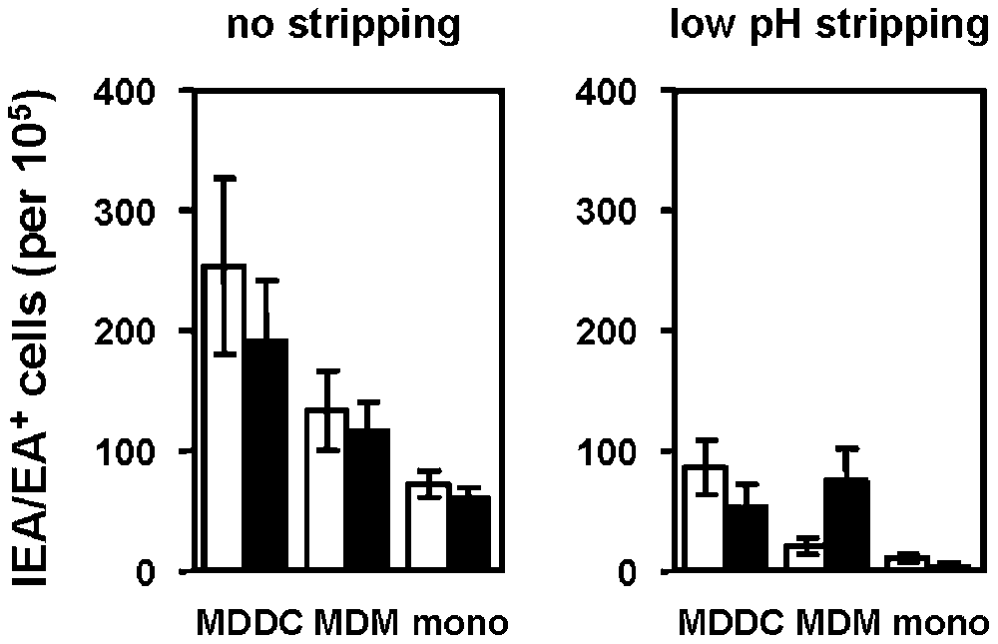
MDDCs can mediate HCMV *trans*-infection through both plasma membrane-associated virions and the release of internalized virions. MDDCs, MDMs or monocytes from the same blood donor were obtained as described in the Materials and Methods section. Cells were pretreated with 40 nM BafA1 (black bars) or the vehicle (DMSO) (white bars) prior to incubation with the VHL/E HCMV strain for two hours (MOI=2). Cells were then extensively washed with a low-pH buffer (glycine 0.2M, pH=2.8) or with PBS alone as indicated above each panel of the figure and were subcultured in close contact with HFFs. After 48 hours, fibroblasts were processed as previously described [14] to evaluate the infection rate due to *trans*-infection by HCMV-loaded cells (absolute number of IE/E antigen-positive fibroblasts among 10^5^ total cells). n= 4 independent experiments with four different donors in total.

Immature DCs have been reported to have a milder endosomal acidification than macrophages and mature DCs, leading to less antigen degradation and processing consistent with the peptide-loading capacities of the MHC molecules [48]. We therefore wondered whether HCMV virions sequestered in the endosomes of MDDCs were protected from degradation. To assess whether the accumulated virions retained the potential to infect, we tried to recover HCMV particles from the endosome-enriched fraction (EE+LE). Unfortunately, we were unable to recover viral particles without inducing irreversible damage to the particles as demonstrated by the absence of infected HFFs when the cells were cultured with the subcellular fractions (data not shown). We therefore used a trans-infection experimental assay that has been previously described [14]. We incubated MDDCs, monocyte-derived macrophages and monocytes from the same blood donor with HCMV for two hours. After infection, the cells were extensively washed in PBS or in a low-pH inactivation buffer and were placed over HFFs. When analyzing the amount of infected HFFs, we noted that all of the tested cells, MDDCs, macrophages and monocytes, allowed the infection of the reporter cells regardless of whether the cells were pretreated with DMSO or bafilomycin A1 (Figure 7, left panel). After the low-pH washes, monocytes were not able to infect HFFs in spite of bafilomycin A1 treatment (Figure 7, right panel), suggesting that most of the effects observed with the PBS washes were due to virions sticking the plasma membrane. Importantly, after the low-pH washes (Supplementary Figure S5), macrophages pretreated with DMSO failed to *trans*-infect HFFs, while the inhibition of endosomal acidification did not impair this *trans*-infection (Figure 7, right panel). This suggests that endosomal acidification may lead to the degradation of viral particles in infected macrophages and confirms our previous results. Pretreatment with bafilomycin A1 followed by the low-pH washes did not abrogate the *trans*-infection of HFFs cells by infected MDDCs (Figure 7, right panel), but tended to reduce it. These results confirm that infected MDDCs likely transmit HCMV particles through close contacts between membrane-bound virions and the plasma membrane of target cells as the primary mechanism of *trans*-infection. Interestingly, these results are in agreement with the work of Falcone and colleagues in which they show that macropinosomes in human DC are unique organelles able to regulate exocytoses [49]. We also showed that after an acidic wash, cells that were free of virions sticking to the plasma membrane were still able to *trans*-infect HFFs and that this ability may be due to the protection of HCMV particles from destruction by the mild and controlled endosomal acidification that characterizes immature DCs.

## Discussion

Human cytomegalovirus can infect and replicate in a broad array of cell types as reflected by the pathology and epidemiology of HCMV diseases. Because dendritic cells have been postulated to have a role in the systemic spread of the virus, we aimed to characterize the mechanisms of HCMV internalization/entry into MDDCs. Indeed, the method of entry of HCMV into cells has been shown to differ between cell types and also between viral strains and according to how they were produced [17], [18], [19]. However, nothing was known about how HCMV enters MDDCs. To that purpose, we used imaging techniques as well as biochemical and functional assays using pharmacological inhibitors to block the successive steps of HCMV infection. Using transmission electron microscopy, we observed open or closed filopodia-like structures in close contact with HCMV particles. We are aware that additional experiments using the 3D focused ion beam-scanning electron microscopy would be required to definitely conclude on the type of cellular structures involved in the CMV entry in MDDCs. Moreover this technology would be also very informative and conclusive in our appreciation of the "in" and "out" status of CMV particles on the TEM images presented in this study. Nevertheless, we observed virions accumulation in vesicle that were usually moderately or even markedly dense to electrons that not only suggest this observed accumulation is located within cells but also that virions accumulate in vesicles of varying densities. The ultrastructural studies presented in this article show HCMV-containing, uncoated vesicles that resemble macropinosomes in term of size (up to 1500–2000 nm in diameter) and content, while others have shown that HCMV entry into human retinal pigment epithelial cells occurs through invagination of the cell membrane with the viral particles coated on the plasma membrane [17]. Dendritic cells engulf antigens into intracellular vesicles in an actin-dependent pathway. Here, we demonstrated that actin polymerisation was indispensable for mediating the engulfment of HCMV into the endocytic compartment of MDDCs. An actin-dependent infection pathway was also observed by others in retinal pigment epithelial and endothelial cells, while human foreskin fibroblast infection has been shown to be independent of actin [17], most likely because HCMV has been shown to enter fibroblasts by direct fusion to the plasma membrane [20]. Cellular factors involved in virus entry by macropinocytosis have been identified for several enveloped viruses (HIV-1, HSV-1 and vaccinia virus), but not for HCMV [16]. In this study, we showed that HCMV entry and subsequent events, i.e., expression of immediate early (IE) and early (E) viral antigens by MDDCs, involve an active, actin-dependent remodeling of the plasma membrane and also depends on Na^+^/H^+^ exchangers, PKC activation and on the integrity of cholesterol-enriched microdomains. We have provided strong evidence to conclude that the HCMV infection process in MDDCs fulfills the criteria that define macropinocytosis as previously defined by others [16]. Our conclusions are consistent with previous studies that reported very similar HCMV endocytic pathways in endothelial cells with another commonly used viral strain TB40/E [50]. We chose to focus our study on VHL/E strain on the basis of in-house data showing that it has often a higher infectious potential toward MDDCs than other entdotheliotropic strains such as TB40/E although the infectious potential may also vary between MDDC preparations for a given strain.

Because the contents of macropinosomes can either be degraded at the late endosome/lysosome stage or recycled at the plasma membrane by a yet unknown mechanism (see [51] and [16] for review; [49]), we analyzed the location of internalized HCMV virions. We observed an accumulation of enveloped HCMV particles in the early endosome compartment. It reached a plateau at six hours post-infection. To lead to a productive infection, the endocytosis of enveloped viruses must be followed by the fusion of the viral envelope and the endocytic vesicle membranes. We have observed uncoated capsids simultaneously close to the nuclear envelope. Interestingly we also noticed a possible association of naked capsids with fiber-like structures we considered as microtubules since microtubule polymerization inhibitors such as nocodazole or colchicin did inhibit IE/E antigen expression by CMV-infected MDDCs (data not shown). Our work is in accordance with papers showing that CMV [52], Kaposi’s sarcoma associated virus [53] and HSV-1 [54] do use the microtubule network to convey their uncoated capsids to the nucleus. Recently, it was clearly shown that in contrast to the requirement for a drop in pH in macrophage endosomes, dendritic cells are able to tune their endosomal pH [48] and to keep it stable by tightly regulating the reactive oxygen species (ROS)/H^+^ balance in endo-lysosomes [55]. In DCs, a stable endosomal pH allows a mild proteolysis that leads to an efficient antigen processing instead of complete protein hydrolysis. We did not observe direct fusion of the HCMV envelope at the MDDC plasma membrane by transmission electron microscopy at rather early time points (30 minutes pi). While a low pH and the UL128-UL150 gene cluster are required to facilitate the penetration stage of the TR strain into epithelial and endothelial cells [18], fibroblasts have been shown to be infected independently of these receptors [56], [57]. This evidence for cell type-specific receptors could explain why HCMV infection of MDDCs is not pH-dependent. VHL/E contains the UL128-UL150 gene cluster and was shown to still be capable of infecting MDDCs. We propose two hypotheses to explain the pH-independent fusion. Firstly, we cannot rule out that our viruses contain heterogeneous particles, some containing the UL128 to UL150 genes and other missing theses genes, which would allow some particles (i.e., those bearing UL128-UL131A gene products) to rapidly enter and further infect MDDCs while others would be internalized but would be unable to promote fusion and thus be prone to accumulation in macropinosome-like vesicles. This hypothesis is in agreement with the results published in a recent paper [58]. Secondly, HCMV virus is known to adapt to its host, and this pH-independent fusion might be another example of its adaptability. It is tempting to postulate that HCMV has evolved to use the endocytic machinery to efficiently penetrate DCs without being entirely destroyed. Further investigation is needed to elaborate on these hypotheses.

Using subcellular fractionation and western blot analyses, we showed that envelope and capsid components, gB and MCP, were still detectable as native full-length proteins in low- and intermediate-density endosomes, most likely early and late EEA1^+^ endosomes. Interestingly, Falcone and colleagues have already described similar EEA1^+^ macropinosome-like vesicles capable of internalizing and concentrating particulate antigens such as latex beads and renamed them enlargeosomes [49]. In addition, qPCR analyses of viral DNA in separated fractions indicated the presence of CMV genomes in all of the tested fractions (Supplementary Figure S3). These observations suggested that the fusion of internalized virions might occur at the late endosome stage in human MDDCs.

We previously demonstrated that DC-SIGN was instrumental for specifically immobilizing HCMV particles at the MDDC plasma membrane, allowing infection. Based on the antibody-mediated neutralization of CMV binding to DC-SIGN, we concluded that this interaction accounts for more than 90% of the binding capacity of MDDCs for CMV[14]. Previous reports have already shown that low-pH buffers (<7) strongly alter the DC-SIGN oligomerization and most probably also its ability to bind with high affinity to its cognate ligands, such as CMV gB [14], [59]. Although it is admitted that acidic washes do inactivate CMV particles that bind to the plasma membrane of fibroblasts or endothelial cells, our observations made with MDDCs provide an alternative explanation for the acidic buffer-mediated inactivation of plasma membrane-stuck CMV particles in our experimental model. Indeed an acidic wash may also promote stripping of CMV virions from outside the MDDCs (Supplementary Figure S5). In this paper, we clearly showed that the stable endosomal pH within the infected MDDCs protects HCMV virions from degradation without impairing MDDC infection. Therefore, the different fates of the macropinosomes described earlier can be observed in the context of HCMV entry into MDDCs, and this leads to both the infection of the cell and the capability for *trans*-infection. Interestingly, a recent paper by Tacken and collegues show that the binding of the neck region of DC-SIGN (using a monoclonal antibody) induces an endocytosis clathrin independant and resulted in a prolonged localization of DC-SIGN in early endosomal compartment. On the other hand, targetting DC-SIGN region with an anti-CDR region lead to the late endosomal compartment [60].

DC-SIGN, either as membrane-associated oligomers or as their soluble counterparts, clearly has a key role in HCMV infection of MDDCs [14], [61]. Located in cholesterol-enriched lipid rafts, DC-SIGN microdomains have been shown to be essential for HIV internalization into MDDCs [42]. Indeed, when cholesterol is depleted from plasma membrane microdomains, the microdomains are disrupted, leading to the disruption of DC-SIGN clusters. The surface expression of DC-SIGN as single molecules and not as clusters is thought to drastically impair high affinity interactions between the lectin and its ligands, leading to a blockage in viral entry [42]. Our data are in total agreement with all these observations because we showed that the destruction of such microdomains using methyl-β-cyclodextrin significantly impaired MDDC infection. We therefore demonstrated that the nanoscaled molecular DC-SIGN clusters located in cholesterol-enriched lipid raft are also essential for HCMV internalization. Because macropinocytosis is cholesterol-dependent, it is tempting to hypothesize that the simple interaction of DC-SIGN with HCMV could be sufficient to induce macropinocytosis via the activation of DC-SIGN-dependent signaling. To our knowledge, this would be the first documented receptor-specific, macropinocytosis-mediated process of virus entry [16]. However, supplemental experiments are needed to determine the importance of this process in the cellular biology of DCs.

HCMV-infected MDDCs have been shown to release infectious HCMV on Day 6 after infection with VHL/E [27]. In this paper, we showed that 48 hours post-infection, while the late antigen pp150 was not even expressed [27], the endosomally protected virions were released after capture and that those particules were remained infectious since they are able to *trans*-infect highly permissive fibroblasts. It appears that HCMV has evolved to use the highly endocytic properties of immature DCs, but not those of monocytes or monocyte-derived macrophages, for its own benefit. Therefore, we propose that besides the latent and replicative viruses, a third pool of CMV might exist as a cell-protected viral pool able to spread all over the body as a "stowaway" taking advantage of the migratory properties of DCs and essentially using the DC as a "Trojan horse" [62]. This observation is not only restrited to CMV infected MDDCs. Interestingly; HIV particles captured by DC-SIGN-expressing cells remain very stable and infectious for several days [63]. Indeed, immature DC-mediated HIV trans-infection has also been shown as being mediated by exocytosis of captured HIV particles. Those exocytosed-particles harbor exosomal antigens [64]. Internalized HCV virus like particles were also protected from lysosomal degradation whithin immature-DC [65] and those infected cell were able to trans-infect Huh-7 cells [66]. In this study, we detected very few particles inside MDDCs up to 24 hours post-infection. Previous work by the group of Gwendalyn Randolph showed that murine DCs are able to migrate from peripheral tissues to lymph nodes within 18 hours. We can assume that upon internalization of infectious CMV particles at entry sites (for example, oro-pharyngeal or ano-genital mucosae), residing DCs may undergo their maturation/migration program, likely leading to the release of still infectious particles in critical locations of the human organism within less than a day [67]. In conclusion, our data indicate that HCMV may have taken advantage of macropinocytosis, a major process to capture antigen, and of the mild endosomal acidification of human immature dendritic cells to allow the protection of HCMV virions while initiating a productive viral infection.

## Supporting Information

Figure S1 **Assessment of the purity of a representative VHL/E stock preparation.** TEM characterization of HCMV preparations is shown. Virions were obtained by ultracentrifugation of end-stage VHL/E-infected human foreskin fibroblast supernatants on a linear tartrate gradient. Picture of one viral stock was obtained by electron microscopy of negatively stained HCMV virions (magnification ×25,000). Black arrows and asterisks indicate intact virions and dense bodies, respectively. A scale bar is indicated in each picture.
(TIF)

Figure S2 **Titration of HCMV genomes by qPCR after subcellular fractionation of HCMV-infected MDDCs.** The absolute number of HCMV DNA copies were quantified in the subcellular fractions of HCMV-infected immature MDDCs (VHL/E; MOI=10) by an in-house qPCR protocol described in the Material and Methods section of this manuscript. The numbers indicated above some columns in the graph represent the indexed values of HCMV DNA copies in comparison to the absolute HCMV DNA copy number (=100) in the postnuclear supernatant (PN). PI means post-infection, and the values represent the absolute number of DNA copies remaining after two hours of incubation. EE=early endosome, LE=late endosome and Ly=lysosome.
(TIF)

Figure S3 **Internalized HCMV virions do not co-localize with LAMP2 whereas a recombinant soluble HCMV gB does. Confocal imaging of particulate (intact HCMV particle) or soluble recombinant HCMV gB in MDDCs.** A) Co-stainings of HCMV gB (red) and LAMP-2 (green) in MDDCs incubated for 15 minutes at 37°C with intact HCMV particles (VHL/E strain; MOI=5; upper row). The results displayed in the lower row show immunostaining (HCMV gB=red and LAMP-2=green) obtained when MDDCs were incubated with soluble recombinant HCMV gB (2 µg/ml; Biomérieux, France) with the same settings reported in A. B) Co-staining of HCMV gB (red) and transferrin receptor (green; AlexaFluor 488-conjugated transferrin) in MDDCs incubated with recombinant soluble HCMV gB. Images were obtained on a SP5 LSM (Leica Microsystems, Germany). DIC images are displayed on the left side of each immunostaining. Single confocal planes are presented. A scale bar is indicated in each DIC picture.
(TIF)

Figure S4 **HCMV internalization into MDDCs is impaired by large spectrum PKC inhibitor.** MDDCs were pre-incubated with rottlerin shown to block PKC activation (rottlerin 40, 13.5, 4.5 µM) and compared to the vehicle (DMSO; 1/100) prior to culturing the cells with virus (VHL/E; MOI=2) for two hours. The cells were then prepared and analyzed as described in the legend for Figure 1D. n= 3 independent experiments with three different donors in total.
(TIF)

Figure S5 **Acidic wash treatment allows for the stripping of HCMV particles from the MDDC plasma membrane.** A) TEM pictures of MDDCs incubated with VHL/E HCMV particles (middle and right panels; MOI=10) or non-infected (left panel). Infected cells were washed with either a low-pH buffer (0.2 M glycine, pH=2.8) or PBS alone and were extensively rinsed before being processed as described in 1A. Black arrows indicate infectious HCMV virions. B) Quantification of infectious HCMV particles by TEM immobilized at the plasma membrane (out, white bars) or internalized into vacuoles (in, black bars) of HCMV-infected MDDCs (two hours with VHL/E; MOI=10) after being washed with a glycine-based acidic buffer (0.2M glycine, pH=2.8; +) or PBS alone (–) (n=10–15 cells per conditions). These results are representative of at least two independent experiments.
(TIF)
