# Correction: Human Cytomegalovirus Entry into Dendritic Cells Occurs via a Macropinocytosis-Like Pathway in a pH-Independent and Cholesterol-Dependent Manner

**DOI:** 10.1371/annotation/d12b8d83-7c45-4661-908d-d7e5a3c9b226

**Published:** 2012-11-29

**Authors:** Fabienne Haspot, Amélie Lavault, Christian Sinzger, Kerstin Laib Sampaio, York-Dieter Stierhof, Paul Pilet, Céline Bressolette-Bodin, Franck Halary

Benoît Rousseau was not included in the author byline. He should be listed as the eighth author and affiliated with Animal facility A2, Université Bordeaux Segalen, Bordeaux, France. The contributions of this author are as follows: Performed the experiments, and analyzed the data.

