# Supplementary figures and images for: Human Cytomegalovirus Entry into Dendritic Cells Occurs via a Macropinocytosis-Like Pathway in a pH-Independent and Cholesterol-Dependent Manner
Source: PLoS One. 2012 Apr 9;7(4):e34795. doi: 10.1371/journal.pone.0034795 (PMC3322158; doi:10.1371/journal.pone.0034795)

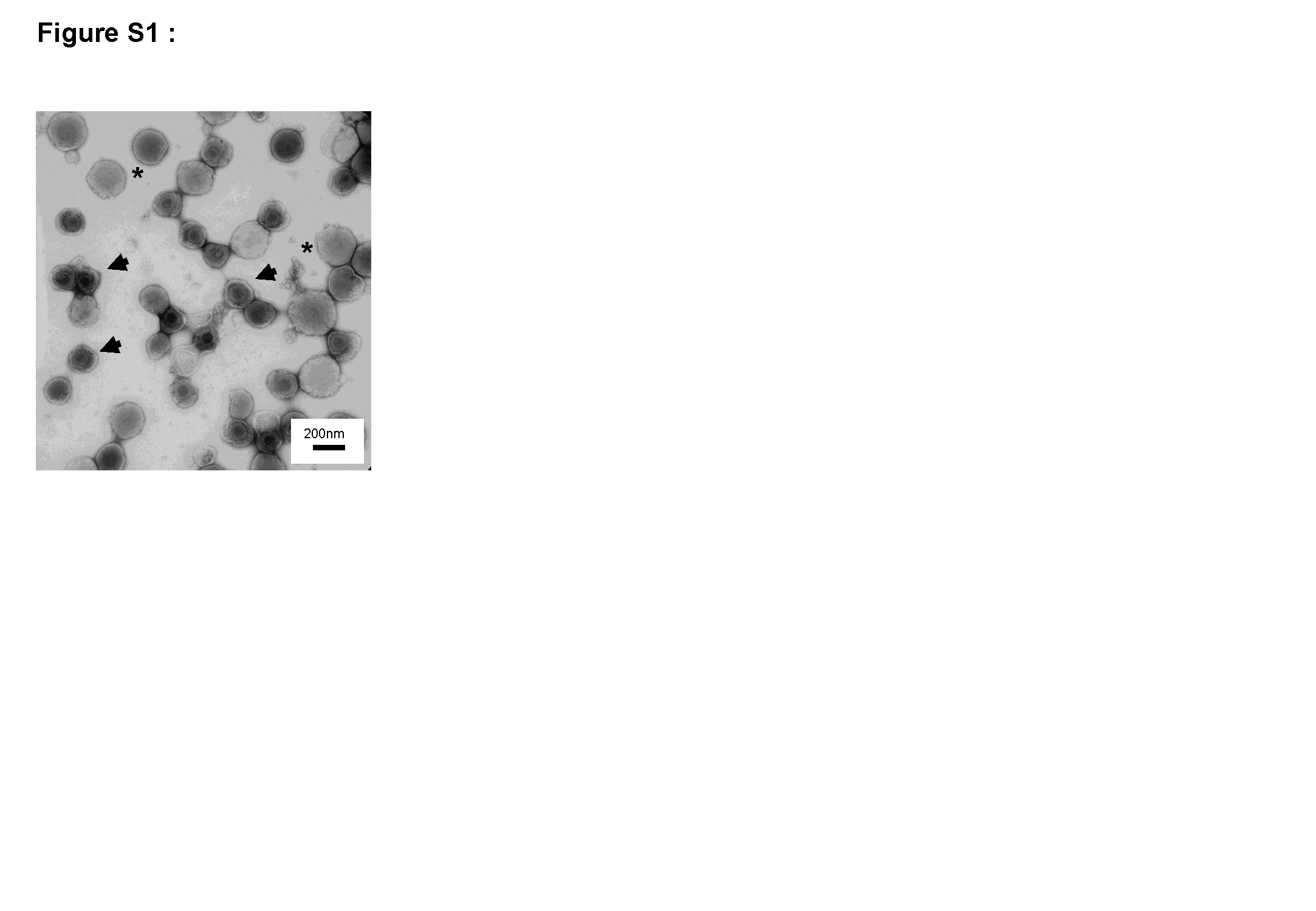

Supplement: Figure S1 — Assessment of the purity of a representative VHL/E stock preparation. TEM characterization of HCMV preparations is shown. Virions were obtained by ultracentrifugation of end-stage VHL/E-infected human foreskin fibroblast supernatants on a linear tartrate gradient. Picture of one viral stock was obtained by electron microscopy of negatively stained HCMV virions (magnification ×25,000). Black arrows and asterisks indicate intact virions and dense bodies, respectively. A scale bar is indicated in each picture. (TIF) [file pone.0034795.s001.tif]

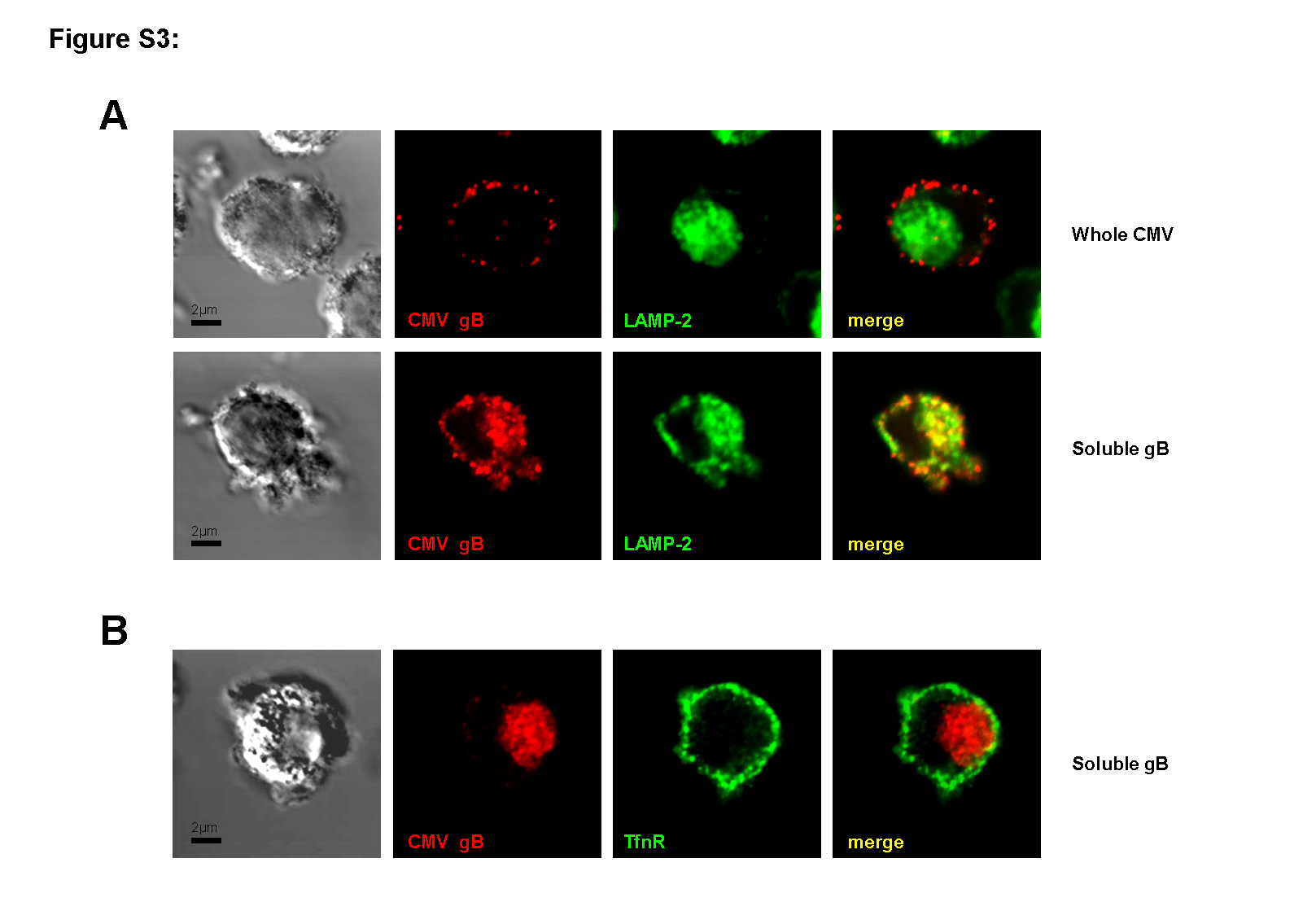

Supplement: Figure S3 — Internalized HCMV virions do not co-localize with LAMP2 whereas a recombinant soluble HCMV gB does. Confocal imaging of particulate (intact HCMV particle) or soluble recombinant HCMV gB in MDDCs. A) Co-stainings of HCMV gB (red) and LAMP-2 (green) in MDDCs incubated for 15 minutes at 37°C with intact HCMV particles (VHL/E strain; MOI=5; upper row). The results displayed in the lower row show immunostaining (HCMV gB=red and LAMP-2=green) obtained when MDDCs were incubated with soluble recombinant HCMV gB (2 µg/ml; Biomérieux, France) with the same settings reported in A. B) Co-staining of HCMV gB (red) and transferrin receptor (green; AlexaFluor 488-conjugated transferrin) in MDDCs incubated with recombinant soluble HCMV gB. Images were obtained on a SP5 LSM (Leica Microsystems, Germany). DIC images are displayed on the left side of each immunostaining. Single confocal planes are presented. A scale bar is indicated in each DIC picture. (TIF) [file pone.0034795.s003.tif]

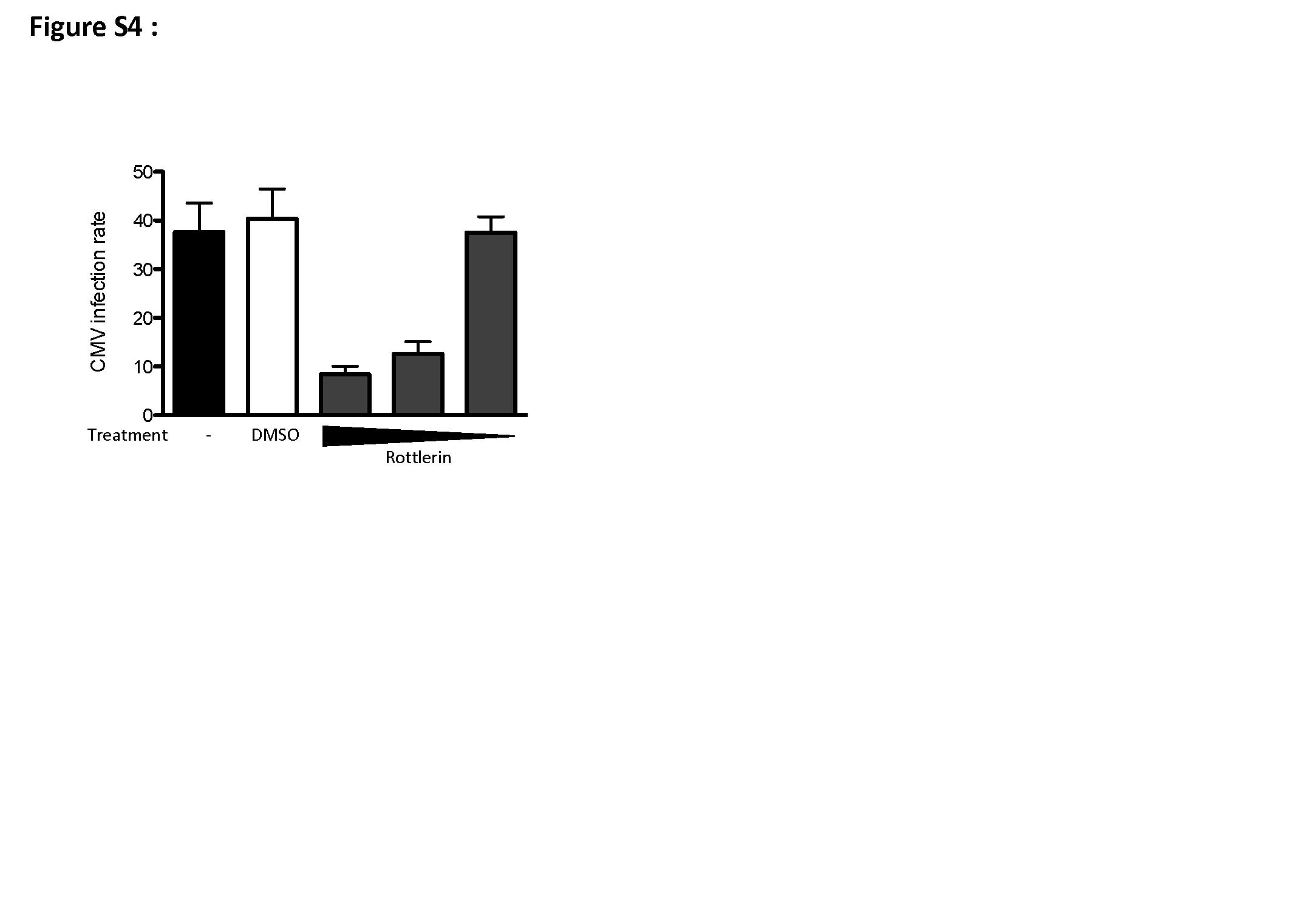

Supplement: Figure S4 — HCMV internalization into MDDCs is impaired by large spectrum PKC inhibitor. MDDCs were pre-incubated with rottlerin shown to block PKC activation (rottlerin 40, 13.5, 4.5 µM) and compared to the vehicle (DMSO; 1/100) prior to culturing the cells with virus (VHL/E; MOI=2) for two hours. The cells were then prepared and analyzed as described in the legend for Figure 1D. n= 3 independent experiments with three different donors in total. (TIF) [file pone.0034795.s004.tif]

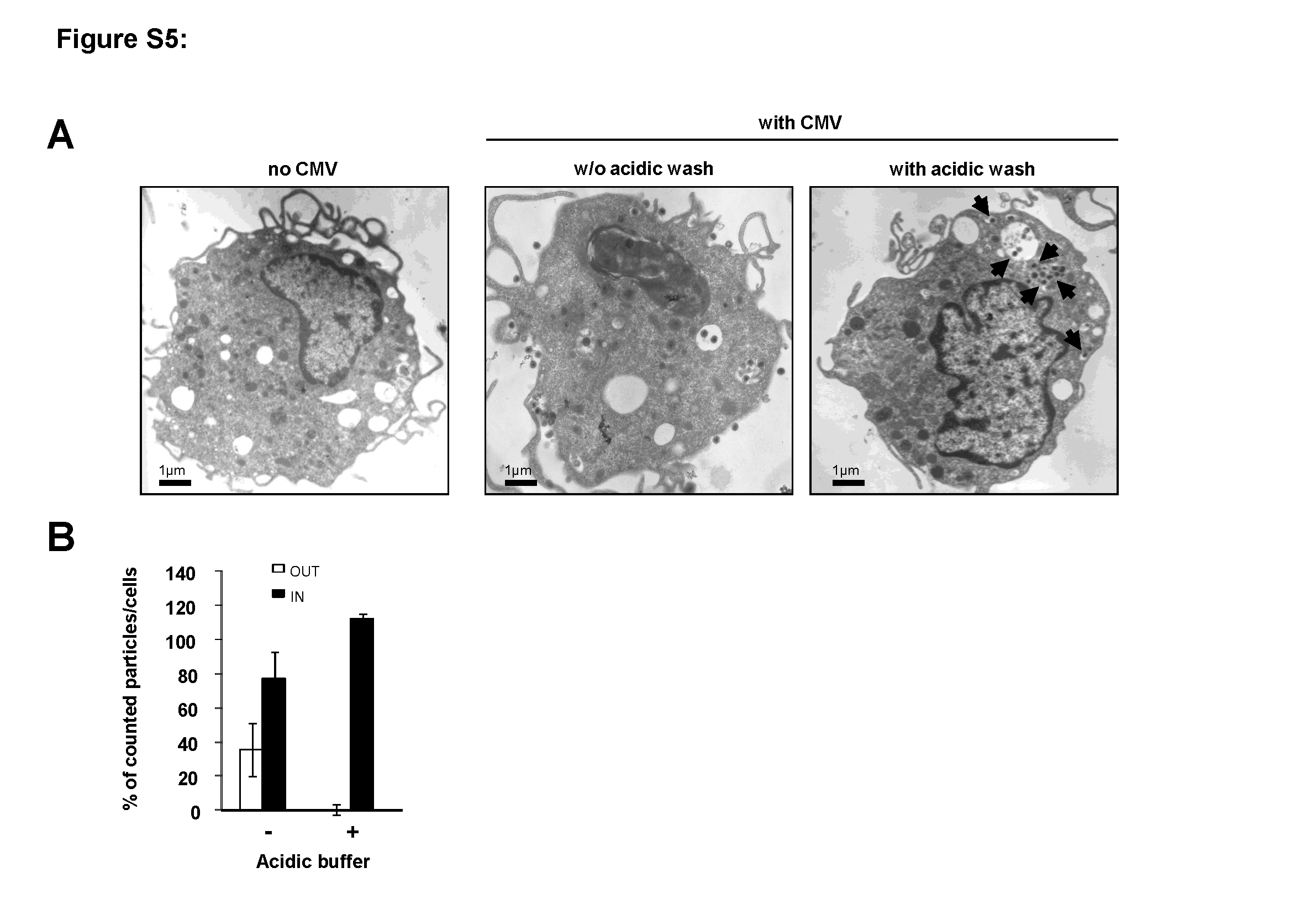

Supplement: Figure S5 — Acidic wash treatment allows for the stripping of HCMV particles from the MDDC plasma membrane. A) TEM pictures of MDDCs incubated with VHL/E HCMV particles (middle and right panels; MOI=10) or non-infected (left panel). Infected cells were washed with either a low-pH buffer (0.2 M glycine, pH=2.8) or PBS alone and were extensively rinsed before being processed as described in 1A. Black arrows indicate infectious HCMV virions. B) Quantification of infectious HCMV particles by TEM immobilized at the plasma membrane (out, white bars) or internalized into vacuoles (in, black bars) of HCMV-infected MDDCs (two hours with VHL/E; MOI=10) after being washed with a glycine-based acidic buffer (0.2M glycine, pH=2.8; +) or PBS alone (–) (n=10–15 cells per conditions). These results are representative of at least two independent experiments. (TIF) [file pone.0034795.s005.tif]
